# Mast Cell Carboxypeptidase A3 Is Associated with Pulmonary Fibrosis Secondary to COVID-19

**DOI:** 10.3390/ijms252212258

**Published:** 2024-11-14

**Authors:** Yatsiri G. Meneses-Preza, Ricardo Martínez-Martínez, Claudia Meixueiro-Calderón, Ulises Manuel Hernández, Elizabeth Angelica Retana, María Dolores Ponce-Regalado, Armando Gamboa-Domínguez, Juan Carlos León-Contreras, Samira Muñoz-Cruz, Rogelio Hernández-Pando, Sonia M. Pérez-Tapia, Alma D. Chávez-Blanco, Enrique Becerril-Villanueva, Rommel Chacón-Salinas

**Affiliations:** 1 Departamento de Inmunología, Escuela Nacional de Ciencias Biológicas, Instituto Politécnico Nacional, ENCB-IPN, Mexico City 11350, Mexico; yatsiri.meneses@gmail.com (Y.G.M.-P.); ricard.martinezmartinez@gmail.com (R.M.-M.); sperezt@ipn.mx (S.M.P.-T.); 2 Departamento de Patología, Centro Médico Naval, Mexico City 04470, Mexico; sofia.meixueiro83@hotmail.com (C.M.-C.); dr.ulisesguevara@gmail.com (U.M.H.); elizabeth.retana@enp.unam.mx (E.A.R.); 3 Departamento de Ciencias de la Salud, Centro Universitario de los Altos, Universidad de Guadalajara, Tepatitlán de Morelos 47620, Mexico; maria.ponce@cualtos.udg.mx; 4 Departamento de Patología, Instituto Nacional de Ciencias Médicas y Nutrición Salvador Zubirán, Mexico City 14080, Mexico; armando.gamboad@incmnsz.mx (A.G.-D.); carlos.leonc@incmnsz.mx (J.C.L.-C.); 5 Unidad de Investigación Médica en Inmunoquímica, UMAE Hospital de Especialidades, Centro Médico Nacional Siglo XXI, Instituto Mexicano del Seguro Social, Mexico City 06720, Mexico; samira.munoz@imss.gob.mx; 6 Sección de Patología Experimental, Departamento de Patología, Instituto Nacional de Ciencias Médicas y Nutrición Salvador Zubirán, Mexico City 14080, Mexico; rhdezpando@hotmail.com; 7 Unidad de Desarrollo e Investigación en Bioterapéuticos (UDIBI), Escuela Nacional de Ciencias Biológicas, Instituto Politécnico Nacional, ENCB-IPN, Mexico City 11340, Mexico; 8 División de Ciencia Básica, Instituto Nacional de Cancerología (INCan, SS), Mexico City 14080, Mexico; celular_alma@hotmail.com; 9 Laboratorio de Psicoinmunología, Instituto Nacional de Psiquiatría Ramón de la Fuente, Mexico City 14370, Mexico

**Keywords:** mast cell, carboxypeptidase A3, COVID-19

## Abstract

COVID-19 is an infectious disease caused by SARS-CoV-2; over the course of the disease, a dysregulated immune response leads to excessive inflammation that damages lung parenchyma and compromises its function. One of the cell lineages classically associated with pathological inflammatory processes is mast cells (MCs). MCs and their mediators have been associated with COVID-19; we previously reported the role of carboxypeptidase A3 (CPA3) in severe COVID-19. However, sequelae of SARS-CoV-2 infection have been poorly studied. In patients who successfully resolve the infection, one of the reported sequelae is pulmonary fibrosis (PF). The etiology and exact mechanisms are unknown, and few studies exist. Therefore, the aim of this study was to evaluate whether MCs are associated with PF development after SARS-CoV-2 infection. Our findings demonstrate that during severe cases of SARS-CoV-2 infection, there is an increased amount of CPA3^+^ MCs in areas with pneumonia, around thrombotic blood vessels, and in fibrotic tissue. Moreover, higher numbers of CPA3-expressing MCs correlate with fibrotic tissue development (r = 0.8323; *p* = 0.001170). These results suggest that during COVID-19, exacerbated inflammation favors the recruitment or expansion of MCs and CPA3 expression in the lungs, which favors tissue damage and a failure of repair mechanisms, leading to fibrosis.

## 1. Introduction

The COVID-19 pandemic has caused more than 7 million deaths worldwide since the discovery of the infectious agent in December 2019 to now. SARS-CoV-2 infection has a broad spectrum of clinical manifestations, with severe forms of the disease being life-threatening. Severe COVID-19 is associated with a dysregulated immune response, where innate immunity is mainly responsible for systemic inflammation and tissue damage [1]. During the COVID-19 pandemic, efforts were focused on fighting the infection, but long-term sequelae were not yet of concern. However, radiographic, spirometry, and laboratory abnormalities have begun to be observed in patients that recovered from severe COVID-19 [2]. Clinical evidence suggests that patients who had severe COVID-19 are at increased risk of having persistent symptoms (post-acute COVID-19 syndrome) or long-term pulmonary sequelae, especially those patients who required oxygen therapy, such as nasal cannulas or mechanical ventilation, during their hospitalization [3,4].

Post-acute COVID-19 syndrome is characterized by persistent clinical manifestations for more than 4 weeks from the onset of symptoms. Some patients experience these symptoms for approximately 4–12 weeks (subacute or ongoing symptomatic COVID-19) or more than 12 weeks (chronic or post-COVID-19 syndrome). Common symptoms include dyspnea, fatigue, joint pain, muscular weakness, chest pain, sleep disturbances, and brain fog [4]. Pulmonary sequelae are the main alterations in patients with post-acute COVID-19 syndrome and include dyspnea, prolonged requirement of supplemental oxygen, and the development of interstitial lung disease (ILD) [5,6]. ILD is a heterogeneous disease group that affects the interstitial tissue compartment of the lungs. Pulmonary fibrosis (PF), one of the diseases that includes ILD, is characterized by impaired repair of damaged lung epithelium, with the persistence of fibroblasts accompanied by the deposition of extracellular matrix (ECM) components, such as collagen, and loss of pulmonary architecture [7]. Post mortem lung studies showed that patients who had a long course of COVID-19, with prolonged hospitalization, had more interstitial fibrosis compared to individuals with shorter disease courses [8]. The exact etiology of COVID-19-associated PF is unknown, but it could be mediated by pneumonia, ARDS, coagulopathy, or immune system dysregulation. The etiology could be considered multifactorial and the result of processes contributing to the breakdown of the endothelial–epithelial barrier, activating tissue repair mechanisms that fail, and promoting a pro-fibrotic state [3,9]. Some cells of the immune system have been implicated in both the regulation of healing processes and the progression to a fibrotic state [10]. Recently, an association has been found between increased mast cells (MCs) in the lungs of COVID-19 patients and the up-regulation of pro-fibrotic genes [11]. Previously, we reported an increase in carboxypeptidase A3 (CPA3) levels in the serum of severe COVID-19 patients, which was a good predictor of disease severity [12]; this suggests that MCs not only participate in SARS-CoV-2 infection pathogenesis but might also be involved in sequelae of post-acute COVID-19 syndrome.

Classically, CPA3 has been considered a marker of MC activation. In healthy individuals, CPA3 is expressed exclusively by MCs, while in atopic patients, it can also be found in basophil granules [13]. CPA3 expression is usually accompanied by the co-expression of tryptase and chymase, so it is speculated that in vivo, the three proteases act together in a coordinated manner to enhance their activity [14]. Despite this and its abundance in MC granules, CPA3 has been less studied than tryptase and chymase. CPA3 is a zinc-dependent metalloprotease with exopeptidase activity, meaning it cleaves proteins after C-terminal amino acid residues, preferentially aromatic or hydrophobic residues. Several substrates of CPA3 have been identified in vitro, including multiple neuropeptides and vasoactive peptides [15]. The population of MCs that store CPA3 appears to be scarce in human lungs. However, although the protein is not stored in granules, its mRNA has been reported to be highly expressed by lung MCs under normal conditions, suggesting constitutive release and a homeostatic function for this protease [16]. Moreover, increased levels of CPA3 have been reported in ILDs, such as idiopathic pulmonary fibrosis (IPF) [17]. Despite the established role of this protease in various pathological processes, its potential involvement in fibrosis resulting from SARS-CoV-2 infection has not been explored. Therefore, this study aims to evaluate the unique CPA3+ mast cell phenotype in the lungs of fatal COVID-19 cases and to investigate its novel association with the development of pulmonary fibrosis (PF).

## 2. Results

### 2.1. Sample and Demographic Data

For this study, fourteen lung tissue samples were obtained from autopsies: eight from patients with COVID-19 and from a control group of six autopsies performed on patients who died prior to the onset of the COVID-19 pandemic. The mean age of the COVID-19 patients was 62.5 ± 11.9 years, of which six (75%) were male and two (25%) were females. The COVID-19 patients had an average hospital stay of 20 days (range 3–48 days) admitted to the intensive care unit. The main comorbidities in our cohort were obesity, hypertension, and diabetes, all diagnosed before hospital admission. No statistically significant differences were found in age, sex, or comorbidities between the COVID-19 patients and the control group, though the small sample size may have limited the detection of subtle differences in these demographics (Table 1).

### 2.2. Morphologic Alterations in Lungs

**Figure 1:**
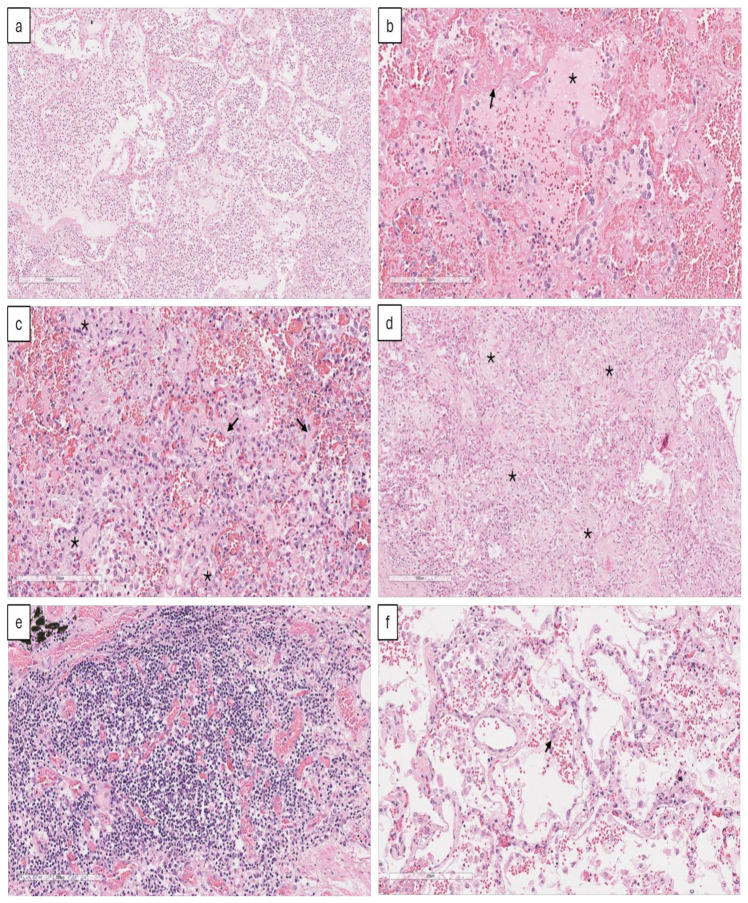
Post mortem histological features in COVID-19-infected lungs. (**a**) During severe COVID-19, pneumonia is characterized by alveolar lumens occupied by extensive leukocyte infiltration. As the infection progresses, lung injury leads to ARDS, whose histologic pattern is diffuse alveolar damage (DAD). DAD has three phases: the first is the exudative phase (**b**), with intra-alveolar proteinaceous eosinophilic material corresponding to pulmonary edema (asterisk), focal intra-alveolar hemorrhage, epithelial cell desquamation, leukocyte infiltration, and hyaline membrane formation (arrow); the proliferative phase (**c**) is characterized by alveolar collapse with hyperplasia of epithelial cells (asterisk) and fibroblasts surrounded by fibrous extracellular matrix producing diffuse interstitial fibrosis (arrow); the last phase of DAD is fibrotic (**d**), in which there is extensive fibrosis (asterisk) and loss of lung structure. Also, during SARS-CoV-2 infection, hemostasis is altered, and there is thrombus formation in small- and medium-sized blood vessels (**e**) and occasional megakaryocytes (arrow) (**f**). Representative micrographs from 8 necropsies of COVID-19, all lung tissue sections stained with hematoxylin and eosin.

The post mortem samples were stained with hematoxylin and eosin to identify histopathological features and areas of interest for the subsequent search of mast cells by immunofluorescence and immunohistochemistry. Diverse levels of tissue damage were found, from areas with initial stages of pneumonia, manifested by abundant inflammatory infiltrate in alveolar spaces with numerous neutrophils, to areas with deposits of eosinophilic fibrillar and amorphous membranes attached to the alveolar walls that are characteristic of diffuse alveolar damage (DAD), indicative of Acute Respiratory Distress Syndrome (ARDS), and middle-sized arteries whose lumen were occupied by thrombi associated with ischemic necrosis that corresponded to infarct areas (Figure 1).

Pneumonic areas showed wide pulmonary interstitium due to edema and infiltration of leukocytes, mainly polymorphonuclear cells and monocytes (Figure 1a); in some patients, epithelial cell desquamation was also observed, accompanying pneumonia.

Areas with different phases of DAD were also observed (Figure 1b–d). Due to ARDS, lesions are not usually uniform, but rather there are areas with free alveolar spaces that alternate with others with alveolar collapse. Histologically, DAD is divided into three progressive phases: exudative, proliferative, and advanced fibrotic phases [18].

The exudative phase (Figure 1b) is an early ARDS stage resulting from damage to alveolar epithelial and capillary endothelial cells. Lung injury triggers an inflammatory response that promotes the infiltration of leukocytes, such as neutrophils and monocytes, which become activated and release mediators capable of contributing to epithelial–endothelial barrier damage. In addition, endothelial damage allows edematous fluid rich in proteins to leak into alveolar spaces, which accumulate, forming hyaline membranes.

The proliferative phase is characterized by the proliferation of fibroblasts and type II pneumocytes, which aim to repair damaged tissue and recover the alveolar lining. In this phase, fibroblast hyperplasia and extracellular matrix deposition are observed as diffuse interstitial fibrosis (Figure 1c).

However, when there is extensive epithelial damage and persistent inflammation, the repair mechanisms fail, resulting in the fibrosis phase, seen as extensive fibrosis that alters pulmonary architecture and impairs normal function (Figure 1d).

In DAD areas, mainly in the exudative phase, it was common to find intra-alveolar hemorrhage and congestion of capillaries and pulmonary blood vessels (Figure 1e) with the presence of megakaryocytes (Figure 1f). Based on these histological findings, we selected three areas of interest for MC quantification: pneumonic, thrombotic, and fibrotic.

### 2.3. Increased CPA3^+^ Mast Cells in Injured Lung Tissue During SARS-CoV-2 Infection

**Figure 2:**
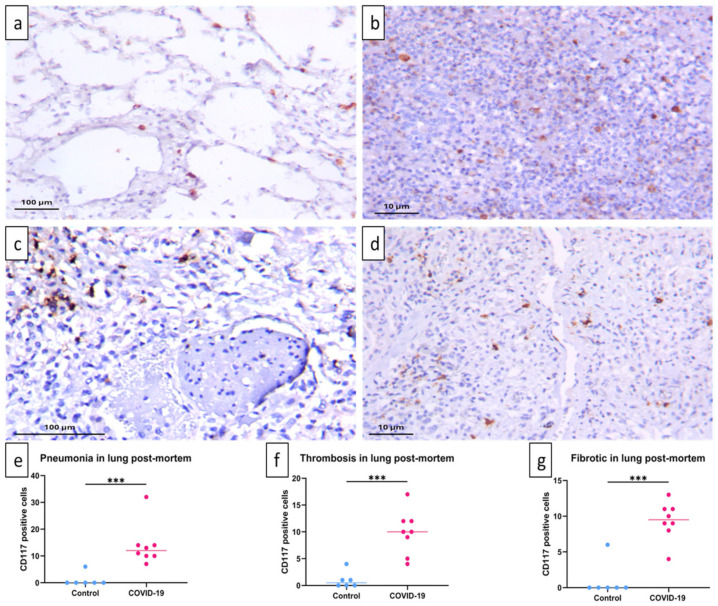
Increase in CD117-positive cells in post mortem lungs of COVID-19 patients. CD117 cells were quantified by immunohistochemical staining of post mortem lung tissue samples. In comparison with control lungs that exhibited CD117 immunostained cells around blood vessels and alveolar capillary interstitium (**a**), lungs of COVID-19 patients showed a significant increase in CD117^+^ cells in areas of pneumonia (**b**) around thrombosed vessels (**c**) and fibrosis (**d**). CD117^+^ nucleated cells were quantified in five fields with distinctive morphology for each individual. Representative micrographs of 6 necropsies of the control group (**a**) and 8 necropsies of COVID-19 patients (**b**–**d**). Median number of CD117-positive cells per patient per field is shown (**e**–**g**). Data are expressed as median ± range and were analyzed with the Mann–Whitney U test (control *n* = 6; COVID-19 *n* = 8) (*** *p* < 0.001).

To identify MCs in histologically injured lung tissue due to SARS-CoV-2 infection, CD117^+^ and CPA3^+^ cells were identified by immunohistochemistry and quantified by automated morphometry. Occasional CD117^+^ cells were found in the control group’s lungs around blood vessels and airways (Figure 2a). In contrast, in COVID-19-infected lungs (Figure 2b–d), CD117^+^ cells were significantly more numerous in areas of pneumonia (Figure 2e) and around thrombotic blood vessels (Figure 2f) and were particularly abundant in fibrotic tissue (Figure 2g).

However, CD117^+^ cells are not only mature MCs and their progenitors but also other hematopoietic progenitor cells recruited from the circulation, as well as innate lymphoid cells [19]. Therefore, we next quantified CPA3^+^ cells in post mortem samples from COVID-19 patients and controls. We chose CPA3 because it is a distinctive protease of MCs. Furthermore, in a previous report, we showed that patients with severe COVID-19 had increased serum CPA3 [12].

**Figure 3:**
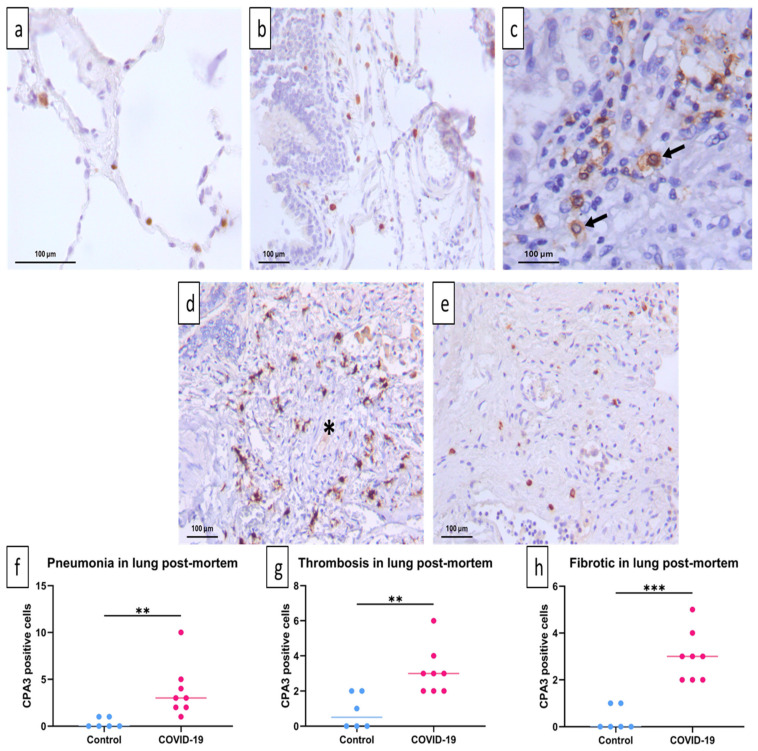
Increase in MC_TC_ in post mortem lungs of COVID-19 patients. CPA3 cells were quantified by immunohistochemical staining of post mortem lung tissue samples. In humans, CPA3^+^ cells belong to the MC_TC_ population and were found in the control group around blood vessels, the alveolar–capillary interstitium (**a**), and the connective tissue below the bronchial epithelium (**b**). In contrast, lungs of COVID-19 patients show a significant increase in MC_TC_ in areas with pneumonia, and some are degranulated (arrows) (**c**), around occluded blood vessels (asterisk) (**d**), and in areas with fibrosis (**e**). CPA3^+^ nucleated cells were quantified in five fields with distinctive morphology for each individual. Representative micrographs of 6 necropsies of the control group (**a**,**b**) and 8 necropsies of COVID-19 patients (**c**–**e**). Median number of CPA3-positive cells per patient per field is shown (**f**–**h**). Data are expressed as median ± range and were analyzed with the Mann–Whitney U test (control *n* = 6; COVID-19 *n* = 8) (** *p* < 0.01; *** *p* < 0.001).

In the control group, CPA3^+^ cells were found throughout the alveolar–capillary interstitial tissue (Figure 3a,b), whereas in COVID-19 patients, these cells were in areas with abundant leukocyte infiltration (Figure 3c), in the wall of thrombotic vessels (Figure 3d), and in areas with fibrosis (Figure 3e). Based on morphology, MCs showed evidence of degranulation in the regions affected by infection (Figure 3c–e) compared with the morphology observed in the control group (Figure 3a,b). The CPA3^+^ cell count showed a significant increase in the total number of MCs in COVID-19 patients compared with the control group in pneumonic (Figure 3f), thrombotic (Figure 3g), and fibrotic areas (Figure 3h) areas. Altogether, these results show an increased number of CPA3^+^ mast cells in COVID-19 patients.

Characteristically, the histological distribution of CPA3^+^ corresponds to the localization of MC tryptase^+^ chymase^+^ (MC_TC_), which is the only type capable of expressing this protease. However, it has recently been described that the MC tryptase^+^ (MC_T_) population, the most abundant in human lungs, expresses high levels of CPA3 mRNA [16] and can increase protease expression during inflammatory processes [20]. Thus, the MC phenotypes are more dynamic and complex than previously thought.

To assess the phenotype of MCs during COVID-19, lung tissue samples from COVID-19 patients and controls were stained by multiplex immunofluorescence for CD117, tryptase, and CPA3. Tryptase and CPA3 are stored in MC granules, which are the main source, and both are considered activation markers. However, some authors have reported their expression in basophils [21]. Therefore, CD117 was also detected because it is a receptor expressed by mature MCs, which is not found in basophils.

**Figure 4:**
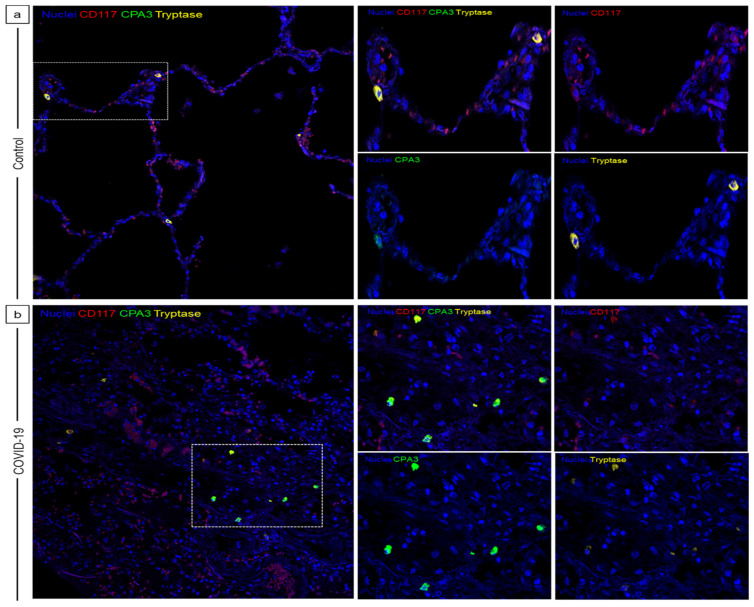
SARS-CoV-2 infection leads to an increase in MC_TC_ in the lungs. Multiple immunofluorescence staining was performed on post mortem lung tissue samples from the control group (**a**) and COVID-19 patients (**b**) (20×). CD117 is shown in red, CPA3 in green, and tryptase in yellow; nuclei were stained with 4′,6-diamidino-2-phenylindole (DAPI). Cells positive for one or both proteases are also positive for CD117, characteristic markers of mature mast cells. The lungs of the control group show few MCs (**a**), whereas in COVID-19 patients, there are more tryptase^+^/CPA^+^ MCs, which belong to the classic MC_TC_ phenotype (**b**). Representative micrographs of 8 necropsies of COVID-19 patients and 6 necropsies of patients who died of non-COVID-19 related causes (control group).

We observed CPA3^−^ MCs (CD117^+^, tryptase^+^, and CPA3^−^) and CPA3^+^ MCs (CD117^+^, tryptase^+^, and CPA3^+^) in both controls and COVID-19 patients (Figure 4). In control group tissues, few CPA3^+^ MCs were found (Figure 4a), whereas in COVID-19 patients, a higher number of CPA3^+^ MCs were observed (Figure 4b). These results indicate that CPA3^+^ cells share molecular markers associated with mast cells.

Altogether, these findings show that during SARS-CoV-2 infection, there is an increase in MCs in the lungs, mainly CPA3^+^ MCs, and these cells are activated and degranulated, which was associated with pneumonia and tissue damage.

### 2.4. Numerous CPA3^+^ Mast Cells in the Lungs of COVID-19 Patients Are Associated with Fibrosis

CPA3^+^ MCs were particularly abundant in pulmonary fibrotic areas of COVID-19 patients (Figure 2g and Figure 3h). Fibrosis is a common sequela of SARS-CoV-2 infection that decreases respiratory capacity and is prevalent in patients who resolve the acute disease [22]. Masson’s trichrome staining was performed to determine lung fibrosis in tissue samples from COVID-19 patients and from controls. The amount of tissue stained by aniline blue (collagen fibers) was then quantified, and the percentage of the fibrotic tissue per field was calculated (% fibrosis).

**Figure 5:**
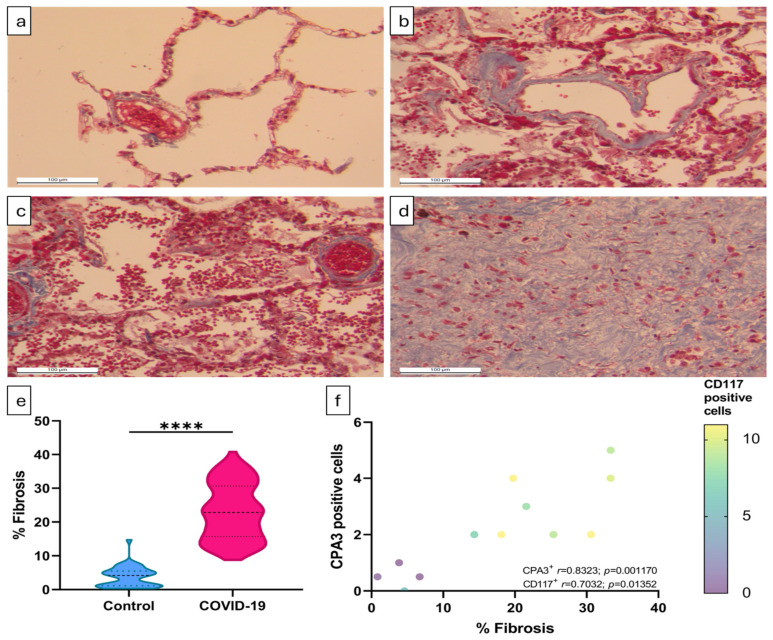
Lung fibrosis of COVID-19 patients correlates with increased MC numbers. Post mortem lung tissue samples from COVID-19 patients and the control group were stained with Masson’s trichrome to measure fibrosis. In the control group, only collagen around blood vessels and airways was observed (**a**). In contrast, COVID-19 lung infection samples show increased collagen deposits in airways and blood vessel walls (**b**) and in the alveolar–capillary interstitium (**c**). Extensive fibrous scar tissue formation is observed in these tissue samples (**d**). The higher percentage of fibrosis in post mortem lungs of patients with COVID-19 was statistically significant. (**e**). Furthermore, it correlates positively and significantly with the number of CD117-positive cells (r = 0.7032; *p* = 0.01352) and the number of CPA3-positive cells (r = 0.8323; *p* = 0.001170). (**f**) For each individual, the percentage of fibrosis was measured in ten randomly taken fields. Representative micrographs of 4 necropsies of the control group (**a**) and 8 necropsies of COVID-19 patients (**b**–**d**). The percentage of fibrosis per field is shown (**e**); data are expressed as median ± range and were analyzed with the Mann–Whitney U test (control *n* = 4; COVID-19 *n* = 8) (**** *p* < 0.0001). The correlation between the percentage of fibrosis and CD117^+^ or CPA3^+^ cells was determined by Spearman’s rank test using the median of fields per individual (**f**).

In the control group, collagen fibers are part of pulmonary connective tissue around bronchioles, blood vessels, and alveolar epithelium (Figure 5a). In contrast, the lungs of patients deceased by COVID-19 showed more deposition of collagen fibers throughout the alveolar–capillary interstitial tissue (Figure 5b) and surrounding blood vessels (Figure 5c). Marked extracellular matrix deposition was also observed substituting the alveolar tissue, corresponding to fibrous scar tissue (Figure 5d). Thus, the percentage of collagen deposition was significantly higher in lung samples from COVID-19 patients compared to the control group (Figure 5e).

The role of MCs in fibrosis development has been associated with their production of pro-fibrotic mediators, such as cytokines, growth factors, and proteases. Hence, we evaluated the correlation between CD117^+^ and CPA3^+^ cells with the percentage of fibrosis in the lungs. We found a positive and statistically significant correlation between the percentage of fibrosis and CD117^+^ cell count (r = 0.7032; *p* = 0.01352), as well as the percentage of fibrosis and number of CPA3^+^ cells (r = 0.8323; *p* = 0.001170) (Figure 5f). These results suggest that MCs may participate in the pathogenesis of COVID-19 by contributing to lung parenchymal inflammation, which may lead to the development of fibrosis.

## 3. Discussion

In this study, we evaluated the presence of MCs in the lungs of fatal COVID-19 cases, finding an increase in the number of CD117^+^ and CPA3^+^ cells in pneumonic, peri-thrombotic, and fibrotic areas. In addition, these MCs showed co-expression of tryptase and CPA3 proteases, suggesting that they belong to the classic MC_TC_ phenotype. These results, taken together, suggest the involvement of MCs in COVID-19 pathophysiology, contributing to inflammation and lung injury.

These results are in line with recent findings of a murine model of SARS-CoV-2 infection, in which human ACE2 (hACE2)-transfected mice, which are MC-deficient, showed decreased leukocyte infiltration, edema, intra-alveolar hemorrhage, and epithelial desquamation compared with the control group, proving that MCs increased lung tissue damage during SARS-CoV-2 infection [23]. Several MC-derived mediators are known for their role in inflammation and increased vascular permeability, such as histamine, leukotrienes (LTC4, LTD4, and LTE4), prostaglandins (PGD2 and PGE2), and platelet-activating factor (PAF); these mediators also contribute to leukocyte recruitment, along with the production of cytokines and chemokines such as TNF-α and IL-8, and PAF has also been associated with platelet aggregation and clot formation [24,25].

The increase in MC numbers observed in the lungs of patients who died from COVID-19 corresponds with previous reports by other authors who evaluated the presence of MCs in lung parenchyma [26,27,28]. However, here we report the tissue distribution of MCs when tissue is altered by SARS-CoV-2 infection. Since pulmonary lesions are often heterogeneous, regions reflecting early stages of infection, such as pneumonia, as well as its progression and vascular alterations with thrombus formation, could be observed. Interestingly, the presence of MCs was also evident at healing sites, indicative of advanced injury and fibrosis. Although research on MC proteases has increased in recent years, most studies have focused on tryptase and chymase, overlooking the role of CPA3.

In this sense, it is important to mention that MC proteases, tryptase, and chymase have been associated with epithelial and endothelial barrier damage through epithelial cell apoptosis and the disruption of endothelial tight junctions [29,30,31]. Additionally, during inflammatory processes, a population of MCs has been described whose phenotype is characterized by the co-expression of tryptase and CPA3, without chymase expression, contrasting with the classic phenotype of MC_TC_. Our findings are consistent with this phenotype, as we observed an increase in CPA3^+^ MCs during SARS-CoV-2 infection and the co-expression of CPA3 with tryptase.

That could indicate that during inflammatory processes, MC_Ts_, which normally reside in the lungs, modify their expression of proteases during inflammatory processes to respond to microenvironment challenges [20]. The reason for the phenotypic shift of these MCs to express CPA3 and the biological function of this protease in pathological conditions is not known. We previously reported increased serum CPA3 levels in severe COVID-19 patients and its association with markers of inflammation during SARS-CoV-2 infection [12]. In summary, these results suggest an unexpected role for CPA3 in pathological hyper-inflammation, such as during severe COVID-19.

Not least is the increase in CD117^+^ and CPA3^+^ cells in the fibrotic areas. Among severe COVID-19 patients who resolve the infection, sequelae due to lung injury may prevail. Epidemiological studies, based on patterns of imaging abnormalities and data from other coronavirus outbreaks, suggest that patients with severe forms of COVID-19 may develop sequelae such as pulmonary fibrosis (PF); this is why the role of inflammation in fibrosis has been described, as well as the participation of immune cells in the release of pro-fibrotic factors [3,10]. Also, this is associated with idiopathic pulmonary fibrosis (IPF) patients who have increased MCs in their lungs, which correlates positively with the degree of fibrosis and negatively with lung function [32,33].

The underlying mechanism for MCs increases in IPF, and its contribution to tissue remodeling processes leading to PF is not entirely clear. However, MC-derived tryptase and chymase have an effect on connective tissue cells involved in fibrosis, such as epithelial cells and fibroblasts, and also on ECM remodeling. For example, tryptase, through the PAR-2 receptor, promotes fibroblast proliferation and type I collagen production [34,35,36], and chymase activates matrix metalloproteinases (MMP)-9 and -2, which promote ECM turnover [37]. Other MC-derived mediators, such as TGF-β, IL-4, and IL-13, are known to play a role in PF [38].

Interestingly, we found that the increase in CPA3^+^ MCs counts correlated positively with PF in COVID-19 patients. Little is known about the role of CPA3 in PF development. In post mortem samples from patients with IPF, Siddhuraj et al. found increased MCs in fibrotic lung tissue; these MCs expressed an increased amount of CPA3 mRNA and stored protein [17]. Multiple substrates of CPA3 have also been described, including neurotensin, kinetensin, leu5-enkephalin [39], neuromedin N, and xenopsin [40]. However, two substrates stand out in the fibrosis context: endothelin-1 and angiotensin. CPA3 can participate in the regulation of endothelin-1 levels and the formation and degradation of angiotensin II [41,42].

Endothelin-1, a well-described pro-fibrotic factor, is a potent fibroblast chemoattractant that promotes their differentiation into myofibroblasts and the production of ECM components [43]. On the other hand, the importance of the angiotensin system in PF development was also demonstrated [44]. Angiotensin II stimulates fibroblast proliferation and thus subsequent ECM deposition [45]; likewise, angiotensin II contributes to alveolar epithelial cell apoptosis, a critical event in the onset of PF [46]. Therefore, CPA3 could indirectly participate in PF development by acting on substrates such as endothelin-1 and angiotensin I. Currently, it is unknown whether there are direct effects of CPA3 on cells involved in fibrosis pathogenesis.

Our study has some limitations due to the small sample size; further studies in larger cohorts are needed. However, our findings demonstrate that there is an increase in CPA3^+^ MCs associated with the pathophysiology of SARS-CoV-2 infection and the development of fibrosis owing to COVID-19. These results add to the growing evidence about the importance of CPA3 in various diseases, which postulates this protease as a promising biomarker and therapeutic target.

## 4. Materials and Methods

### 4.1. COVID-19 Tissue Processing

This study included eight lung biopsies from patients who died during May 2020 with a confirmed diagnosis of SARS-CoV-2 by RT-PCR testing. Tissues were obtained within 3 h after death. All tissue samples were processed under standard BSL-2 (biosafety laboratory) measures in the Department of Pathology and immediately fixed with 10% neutral-buffered formalin for at least 24 h for preservation and subsequent inclusion in paraffin. The control group consisted of six lung samples from necropsies without morphological alterations associated with viral or bacterial infections; the sample selection period was prior to the onset of the pandemic.

The inclusion criteria were as follows: diagnosis of COVID-19 by RT-PCR and signed authorization for use of biological samples.

The exclusion criteria were as follows: individuals with immunosuppressive treatments; individuals infected with HIV; individuals with oncological, autoimmune, or allergic pathologies; pregnant women; and individuals whose family members decided not to sign the authorization.

The elimination criteria were as follows individuals whose samples were unsuitable for analysis, with incomplete clinical data, or with elimination requested by a legal family member.

### 4.2. Histology and Immunohistochemistry

Three-micron-thick sections were obtained and mounted on poly-L-lysine-coated slides. Then, the sections were deparaffinized, rehydrated, and stained with hematoxylin–eosin (H&E) and Masson’s trichrome staining methods. Images of the whole sample stained with H&E were obtained using Aperio CS2 (Leica Biosystems, Wetzlar, Germany) with 10× and 20× objectives. For the selection of areas of interest, images were exported using eSlide Scan Scope v12.3.3 software (Leica Biosystems, Wetzlar, Germany).

Micrographs of samples stained with Masson’s trichrome were acquired using a Leica DMLS microscope with a 20× dry objective equipped with a Leica DFC295 camera and analyzed with Leica Application Suite (LAS) Version 4.9.0 software (QWin Leica; Wetzlar, Germany). For each patient and control, ten random fields were acquired. Finally, color deconvolution was performed to quantify the extent of fibrosis, expressed as a percentage, by the quotient of the collagen (blue tissue) area divided by the total tissue area scanned per field.

Tissue samples from COVID-19 autopsy cases and controls were stained by immunohistochemistry to detect MCs. Briefly, antigen retrieval was performed by immersing the slides in antigen retrieval buffer (DV2200SC23, Diva Decloaker, Biocare Medical, Pacheco, CA, USA) for 25 min in boiling water. Endogenous peroxidase activity was inhibited by peroxide block (CMC925660030, Cell Marque, Merck KGaA, Darmstadt, Germany) for 20 min; the slides were incubated for 2 h at room temperature with antibodies anti-CD117 (c-kit) (clone: YR145, Cat. 117r-16, Cell Marque, Merck KGaA, Darmstadt, Germany) and anti-CPA3 (Carboxypeptidase A3) (polyclonal ab251685 Abcam, Cambridge, UK). After three washes, tissue was processed with a HiDef Detection™ HRP Polymer System (CMC954080040, Cell Marque, Merck KGaA, Darmstadt, Germany) for 30 min at room temperature and revealed with DAB substrate (CMC957080031, Cell Marque, Merck KGaA, Darmstadt, Germany) for 10 min. Finally, sections were counterstained with hematoxylin and mounted in resin. Micrographs were acquired using a Leica DMLS microscope with 2.5× and 40× dry objectives equipped with a Leica DFC295 camera and analyzed using an automated image analyzer (QWin Leica; Wetzlar, Germany).

For the quantification of CD117^+^ or CPA3^+^ cells, for each COVID-19 patient, 15 fields were acquired with morphology corresponding to 5 pneumonic areas, 5 peri-thrombotic vessel areas, and 5 fibrosis areas, while in samples from the control group, 10 fields were acquired to compare with selected areas of COVID-19-injured tissue: alveolar–capillary interstitial areas for comparison with pneumonia and interstitial fibrosis and the adventitia layer of medium-sized arteries to compare with peri-thrombotic areas.

### 4.3. Immunofluorescence

Slides were heated in a bench-top oven at 70 °C for 45 min to remove excess paraffin. Then, tissue sections were rehydrated in serial solutions of 100% Xylene, Xylene/Ethanol 50/50 (*v*/*v*), 80% Ethanol, 50% Ethanol, and deionized water. Antigen retrieval was performed in citrate buffer pH 6.0 (sodium citrate 10 μM) at 90 °C for 20 min. Tissue sections were permeabilized for 2 h in blocking solution (bovine serum albumin 10 mg/mL, horse serum 5%, Triton 0.3%, and sodium azide 0.02%) and incubated overnight with the following primary antibodies: anti-CPA3 (polyclonal ab251696 Abcam, Cambridge, UK), anti-CD117 (polyclonal PA5-143073 Invitrogen, Thermo Fisher Scientific, Waltham, MA, USA), and anti-tryptase (clone AA1 Abcam, Cambridge, UK). Tissues were washed five times with PBS and then incubated for 2 h with the following secondary antibodies: donkey anti-rabbit IgG (H + L)-Alexa Fluor 488, donkey anti-goat IgG (H + L)-Alexa Fluor 594, and donkey anti-mouse IgG (H + L)-Alexa Fluor 647, all from Jackson ImmunoResearch (West Grove, PA, USA). The stained tissues were counterstained with DAPI for 5 min, mounted with VECTASHIELD antifade mounting medium (Vector Labs, Burlingame, CA, USA), and examined with an Eclipse Ti inverted confocal microscope (Nikon Corporation, Tokyo, Japan) using NIS Elements v.4.50. Imaging was performed using a 20× (dry, NA 0.8) objective lens. Zoom was performed at 3.4×. Images were analyzed using FIJI ImageJ1 Software (National Institutes of Health, Bethesda, MD, USA).

### 4.4. Statistical Analysis

Data were analyzed using GraphPad Prism v9 for Windows software. Data normality was assessed by the Kolmogorov–Smirnov test with the Dallal–Wilkinson–Lilliefor corrected *p*-value. Comparison of the two groups was performed with the Mann–Whitney U test. Statistically significant differences between groups were marked with asterisks (** *p* < 0.01; *** *p* < 0.001; **** *p* < 0.0001). Spearman’s rank correlation coefficient was used to determine the correlation between two variables. The coefficient of variation (CV) of the number of CD117^+^ cells, the number of CPA3^+^ cells, and the percentage of fibrosis per patient or control was calculated. The median was chosen as a representative measure per patient for comparisons between the two groups and to determine the correlation between two variables (CV > 20%).

## Figures and Tables

**Table 1:** Demographic characteristics of COVID-19 patients and control group.

	Control	COVID-19	
*n*	6	8	*p*-Value
Age (Mean ± SD)	55.33 ± 28.42	62.5 ± 11.90	0.5291
Gender, male (%)	2/6 (33.33%)	6/8 (75%)	0.2774
Gender, female (%)	4/6 (66.67%)	2/8 (25%)	0.2774
Overweight (%)	1/6 (16.67%)	3/8 (37.5%)	0.5804
Hypertension (%)	2/6 (33.33%)	2/8 (25%)	>0.9999
Diabetes (%)	2/6 (33.33%)	2/8 (25%)	>0.9999
Smoker (%)	0/6 (0%)	2/8 (25%)	0.4725
COPD (%)	0/6 (0%)	2/8 (25%)	0.4725
Days of intubation * [Median (Q1–Q3)]	-	20 (3–48)	-

* All intubated patients were admitted to the intensive care unit on the same day. Data are reported as median (IQR), mean (± SD), and *n* (%). Comparisons were performed with Student’s *t*-test or the Mann–Whitney U test.

## Data Availability

The original contributions presented in the study are included in the article/Supplementary Materials. Further inquiries can be directed to the corresponding authors.

## Supplementary Materials

The supporting information can be downloaded at: https://www.mdpi.com/article/10.3390/ijms252212258/s1.
